# Exploring aromatic cage flexibility of the histone methyllysine reader protein Spindlin1 and its impact on binding mode prediction: an in silico study

**DOI:** 10.1007/s10822-021-00391-9

**Published:** 2021-06-03

**Authors:** Chiara Luise, Dina Robaa, Wolfgang Sippl

**Affiliations:** Institute of Pharmacy, Martin Luther University of Halle-Wittenberg, Kurt-Mothes-Str.3, 06120 Halle/Saale, Germany

**Keywords:** Spindlin, Histone reader proteins, Pocket flexibility, Molecular dynamics simulation, Induced fit docking, Epigenetics

## Abstract

Some of the main challenges faced in drug discovery are pocket flexibility and binding mode prediction. In this work, we explored the aromatic cage flexibility of the histone methyllysine reader protein Spindlin1 and its impact on binding mode prediction by means of in silico approaches. We first investigated the Spindlin1 aromatic cage plasticity by analyzing the available crystal structures and through molecular dynamic simulations. Then we assessed the ability of rigid docking and flexible docking to rightly reproduce the binding mode of a known ligand into Spindlin1, as an example of a reader protein displaying flexibility in the binding pocket. The ability of induced fit docking was further probed to test if the right ligand binding mode could be obtained through flexible docking regardless of the initial protein conformation. Finally, the stability of generated docking poses was verified by molecular dynamic simulations. Accurate binding mode prediction was obtained showing that the herein reported approach is a highly promising combination of in silico methods able to rightly predict the binding mode of small molecule ligands in flexible binding pockets, such as those observed in some reader proteins.

**Supplementary Information:**

The online version contains supplementary material available at 10.1007/s10822-021-00391-9.

## Introduction

Histone reader proteins are components of a large family of proteins that regulate epigenetic activity by binding to specific histone tails. They are able to recognize posttranslational modifications (PTMs) like methylation, acetylation and phosphorylation, and upon the histone binding, they recruit components of the transcriptional machinery and chromatin remodeling complexes. In addition, posttranslational modifications on non-histone proteins can be also recognized by the reader proteins [1–4]. Extensive research in epigenetic mechanisms has highlighted that PTMs mechanisms are involved in the genesis and development of diverse human diseases, most importantly cancer and neurodegenerative diseases [5].

Spindlin1 is a chromatin reader protein that comprises three Tudor domains and it is known to recognize two different histone marks, H3K4me3 (H3 trimethylated at lysine 4) and H4K20me3 (H4 trimethylated at lysine 20) [6–10]. The latter interaction has been discovered later and, hence, it has been less investigated. A study has also suggested that the H4K20me3 mark may act as a secondary substrate for Spindlin1 because it shows a weaker affinity compared to H3K4me3 [10]. Among the three Tudor domains of Spindlin1, the second domain is well known to bind to the trimethylated lysine marks (K4me3 and K20me3) on the histone tails and to small molecules inhibitors [8, 11, 12]. Instead, the first domain has been reported to recognize asymmetrically demethylated arginine residues (Rme2a) and positive nitrogen moieties of bivalent inhibitors, which simultaneously bind to the first and second domains [8, 11, 13]. Interestingly, the presence of Rme2a on the histone tail has been shown to have opposite effects on the histone peptide affinity: it has been reported to increase the affinity of H3K4me3 (H3K4me3R8me2a) and to decrease the affinity of H4K20me3 (H4K20me3R23me2a) [8, 10]. Furthermore, in a very recent study, it has been reported that Spindlin1 recognizes the bivalent methylation pattern H3K4me3K9me3/2, and specifically, the binding of K9me3/2 to the first domain has been shown to enhance the histone binding affinity [14]. Spindlin1 has been connected to several types of malignant tumors such as ovarian cancer, non-small-cell lung cancers, breast cancer and triple negative breast cancer, liposarcoma and only recently to liver cancer [15–20]. It has also been suggested that Spindlin1 may play a role in tumorigenesis [21–25].

**Fig. 1:**
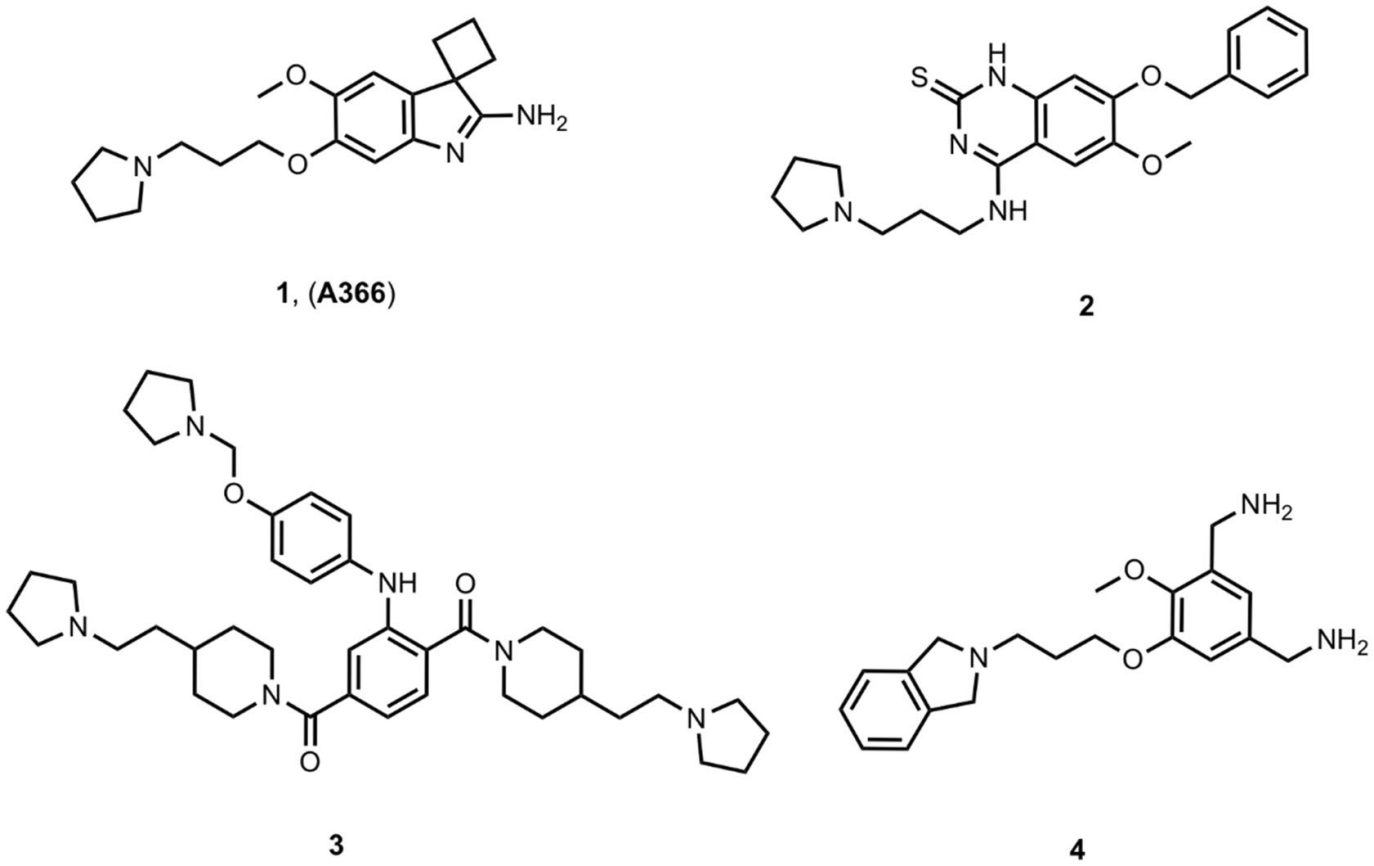
2D structures of reported Spindlin1 inhibitors

Due to the therapeutic potential of Spindlin1 inhibitors, a growing interest has arisen around this target. Hence, several inhibitors have been identified in recent years, and some of them are reported in (Fig. 1). Initially, 1 (A366)—a previously reported G9a inhibitor (IC50: 3.3 nM, [26])—was discovered through a screening platform to also be a Spindlin1 nanomolar inhibitor (IC50: 186.3 nM, [27]). By means of in *silico* studies combined with synthesis and in vitro testing, we later identified novel Spindlin1 inhibitors active in the low µM range (compound 2 is shown in Fig. 1) [28]. Some groups also reported on the development of bivalent inhibitors [11, 13], including compound 3 (Kd of μM) that is shown in Fig. 1 [13]. Moreover, other small molecules inhibitors that inhibit Spindlin1 through binding to the second domain have been described [11, 12], such as the nanomolar inhibitor 4 (Fig. 1).

In the last years, several Spinldin1 crystal structures have been released in the PDB [29], which highlighted that the aromatic cage, responsible for the binding of trimethylated lysine and mimetic moieties, can undergo conformational changes. It is worth noting that aromatic pocket flexibility was also observed in other reader proteins like CBX7, TDRD3 and 53BP1 tandem Tudor domain [30–32]. A comprehensive analysis of the aromatic-cage-containing crystal structures deposited in the PDB revealed that aromatic pockets are observed in diverse protein classes, such as transcription regulators (mainly histone methylation reader proteins), signaling proteins and hydrolases [33]. We can thus speculate that aromatic cage flexibility is present in other non-reader proteins too and evaluating it can be relevant in structure-based studies.

In silico approaches that do not take protein flexibility into account could have limitations in exploring the binding mode of novel compounds for which no crystal structures have been resolved. Indeed in cases where significant changes in the conformation of the binding pocket occur, rigid-body docking is generally not suitable to investigate the binding mode of compounds since the protein is treated rigidly.

In this work, we set to assess the ability of various in silico methods to rightly reproduce the binding mode of known ligands in Spinlin1, as an example of a reader protein showing flexibility in the methyllysine binding pocket. Specifically, we first explored the binding pocket plasticity through molecular dynamics (MD) simulation. Then, we used the cocrystallized inhibitor A366 to probe the ability of induced fit docking (IFD) to reproduce the experimentally determined binding mode with the aim to test if the right ligand binding mode can be obtained through flexible docking regardless of the initial protein conformation. Finally, the stability of generated docking poses was verified by MD.

## Results and discussion

### Analysis of available protein crystal structures

**Fig. 2:**
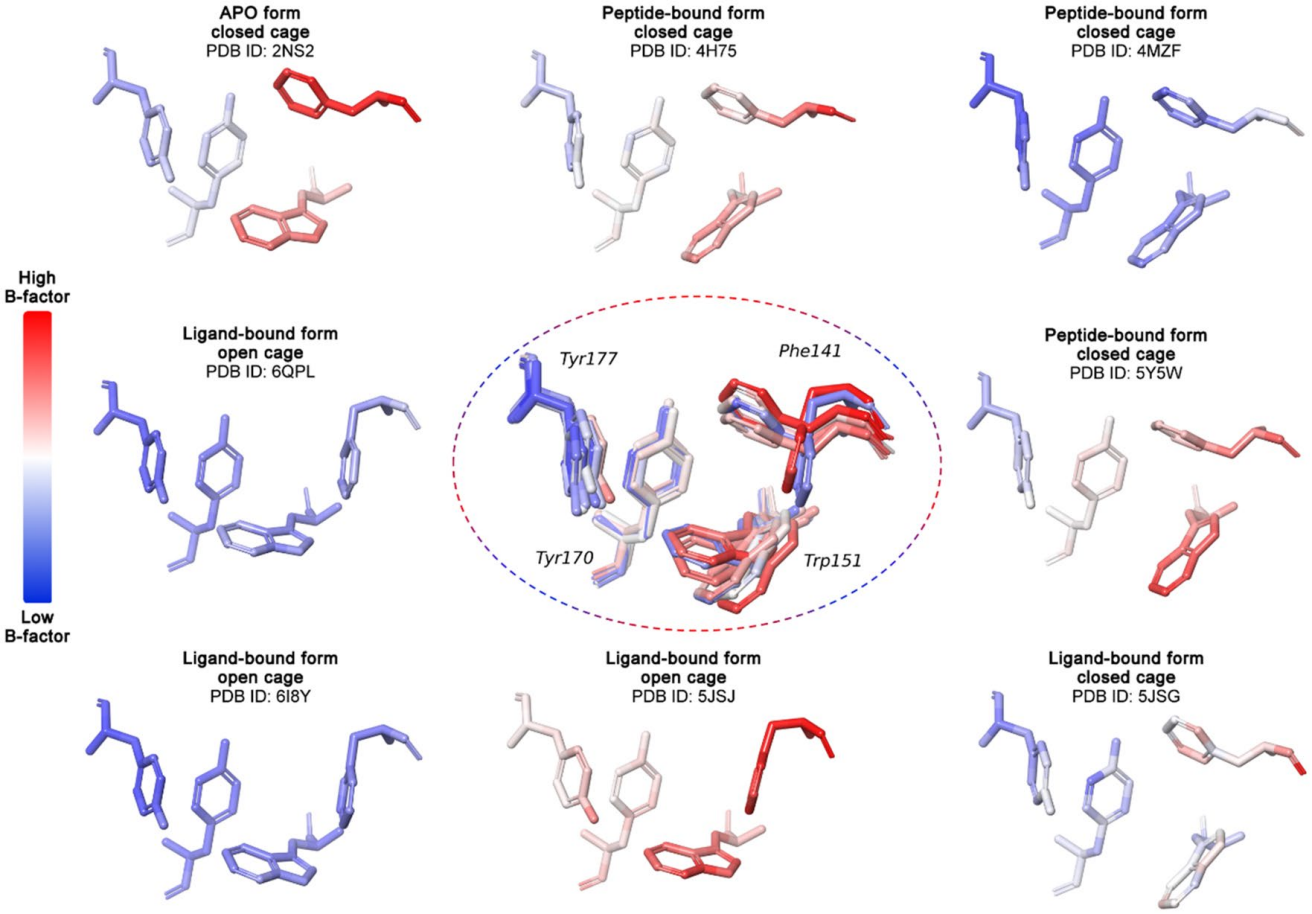
Superimposition and comparison of the aromatic cage of the investigated Spindlin1 crystal structures, PDB IDs: 2NS2, 4H75, 4MZF, 5Y5W, 5JSG, 5JSJ, 6QPL, 6I8Y. The four aromatic amino acids of the aromatic cage are displayed as sticks, and they are colored according to the PDB B-factor values

As a first step, the Spindlin1 crystal structures deposited in the Protein Data Bank (PDB) [29] were analyzed in order to investigate the conformational flexibility of the binding pocket residues. Attention was given to the second domain and specifically to the aromatic cage residues (Phe141, Trp151, Tyr170, Tyr177) as it is responsible for the recognition of the trimethylated lysine and mimetic moieties like the positively charged pyrrolidine. The binding sites of the PDB structures were aligned and the protein residues were colored by PDB B-factor. Several X-ray structures are depicted in Fig. 2 as examples of the different binding pocket conformations that were observed (PDB IDs: 2NS2, 4H75, 4MZF, 5Y5W, 5JSG, 5JSJ, 6I8Y, 6QPL [7, 8, 10–13, 34]). The analysis highlighted that among the four residues of the aromatic cage, Phe141 and Trp151 show higher temperature factor values reflecting uncertainty in the positions of their atoms in the protein crystal structures, hence indicating a higher degree of flexibility of these two amino acids. The side chain of Phe141 can adopt two different orientations which lead to two different shapes of the aromatic cage: either a closed cage (Fig. 2, PDB IDs: 2NS2, 4H75, 4MZF, 4MZH, 5JSG, 5Y5W) or an open cage (Fig. 2 PDB IDs: 5JSJ, 6QPL, 6I8Y). The open conformation is observed only in the ligand-bound forms, except in the crystal structure of Spindlin1 with the bivalent inhibitor EML405 (PDB ID: 5JSG) where Phe141 shows a closed conformation. This suggests that the ligands can generally induce the flip of the Phe141 side chain. Contrariwise, in the apo-form and peptide-bound crystal structures, the side chain of Phe141 adopts the closed conformation. Moreover, Trp151 displays slightly different orientations among the crystal structures to better interact with the positively charged moieties of the co-crystalized ligand/peptide. Additionally, the B-factor values underlined that even in the presence of the ligand or peptide, Phe141 and Trp151 can have a low empirical electron density and, thus, their position is less clearly defined.

### Molecular dynamics simulation of apo-form structure

To further evaluate the flexibility of the aromatic cage as well as of the other binding site residues, and to test whether it is possible to obtain the open conformation starting from the closed conformation, the apo-form crystal structure (PDB ID: 2NS2) was subjected to 50 ns MD simulation using Desmond package [34, 35].

**Fig. 3:**
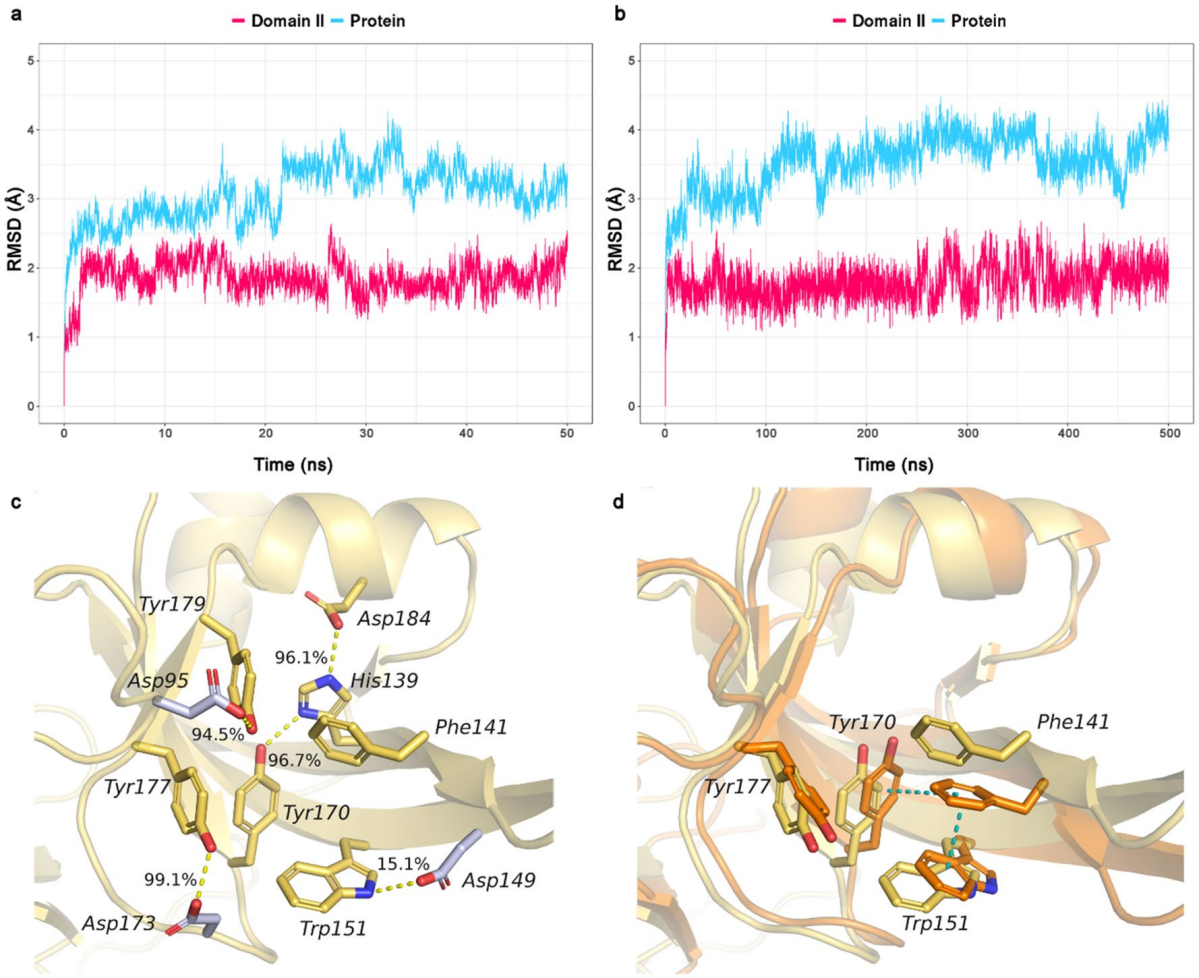
Analysis of 50 ns (**a**, **c**, **d**) and 500 ns (**b**) MD simulation of the apo-form Spindlin1 (PDB ID: 2NS2). **a**, **b** Root mean square deviation (RMSD) plots of backbone atoms. **c** Binding pocket residues (yellow sticks) and hydrogen bonds (yellow dashed lines) as observed in the crystal structure; occupancy values of the interactions during the MD simulation. **d** Superimposition of the reference X-ray structure (yellow, PDB ID: 2NS2) and a representative frame of the new closer cage conformation (orange)

**Fig. 4:**
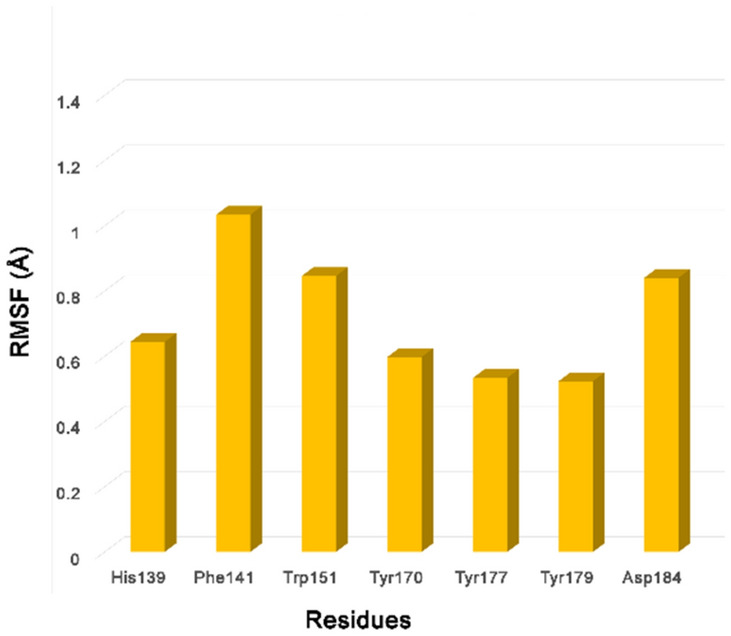
Analysis of 50 ns MD simulation of the apo-form Spindlin1 (PDB ID: 2NS2). Root-mean-square fluctuation (RMSF) values of the binding pocket residues, heavy atoms

The root-mean-square deviation (RMSD) plots (Fig. 3a) showed that while the protein backbone atoms of the whole protein show a relatively high RMSD fluctuation of 2.5–4 Å, the second domain remains rather stable throughout the simulation (RMSD < 2.5 Å). We then focused our attention on the binding pocket and analyzed the RMSF (root-mean-square fluctuation) values of the heavy atoms of specific amino acids that constitute the pocket (Fig. 4). A closer look at those residues revealed that some amino acids are quite steady (His139, Tyr170, Tyr177, Tyr179). On the other hand, Phe141 and Trp151 show higher fluctuations (RMSF 1 and 0.8 Å, respectively) confirming what was already observed before. Nevertheless, the deviations of the latter two residues are still small. Of note, the high RMSF values of Asp184 can be attributed to the flip of its carboxyl group.

The stability of most of the binding site residues can be explained by the hydrogen bond networking established in the pocket. Indeed, hydrogen bonds are formed among the amino acids which contribute to stabilizing their side chains. In Fig. 3c, the binding pocket residues and the hydrogen bonds observed in the crystal structure (PDB ID: 2NS2) are shown. To corroborate the assumption that the hydrogen bond network contributes to the stability of the binding pocket residues, we analyzed the occupancy of these interactions during the MD simulation; values are detailed in Fig. 3c. It was observed that the majority of the hydrogen bonds are preserved during the simulation (occupancy rates grater then 94%) and they can, hence, play a role in stabilizing some of the pocket residues. Only the interaction between Trp151 and Asp149 shows low occupancy rate (15.1%). Giving the nature of Phe, no hydrogen bond interaction can be formed that could stabilize its side chain.

Most interesting is that the open cage conformation was not observed at any instance during the MD simulation time of 50 ns. Instead, as detected during the MD simulation, pi-pi interactions between Phe141 and Trp151 are established leading to a more closed pocket. In fact, after circa 3 ns, the side chain of Phe141 rather moves toward Trp151 and the orientation of these two aromatic residues is mostly stabilized by face-to-face pi-pi stacking interactions. Edge-to-face stacking interactions between Phe141 and Tyr170 are also observed during the simulation but to a much lesser degree. Therefore, Phe141 does not flip during the simulation to generate the open cage conformation but it rather goes closer into the cage. In Fig. 3d is depicted the superimposition of the reference X-ray structure (PDB ID: 2NS2) and a representative frame of the new closed cage conformation. To check the different Phe141 orientations retrieved during the simulation and to quantify their occupancy, the trajectory was clustered based on the RMSD of Ph141. The clustering analysis provided further evidence that the more closed conformation is predominant during the simulation, showing an occupancy rate of 82.2%. A total of three clusters were attained which highlighted that Phe141 and Trp151 mainly move closer as they undergo pi-pi stacking interactions. A representative frame for each cluster and their occupancy rates, as well as the reference X-ray structure, are reported in Figure S1.

We carried out a second extended MD simulation (500 ns) in order to test whether other aromatic cage conformations can be observed during longer simulation time. However, the analysis of the simulation confirmed the same trend observed in the shorter simulation (50 ns). The protein backbone atoms are stable, with relatively higher fluctuation for the whole protein (RMSD 2.5–4.5 Å) and a rather stable second domain (RMSD < 2.7 Å), Fig. 3b. Among the binding site residues, Phe141 and Trp151 still show the highest RMSF values (Figure S2b). Clustering of the trajectory based on the RMSD of Ph141 resulted in a greater number of clusters (16 clusters) as compared to the shorter simulation. Nevertheless, in the vast majority of the clusters, Phe141 and Trp151 still exhibit a face-to-face pi-pi stacking. In Figure S2d a representative frame for each of the first four most populated clusters is shown, while the occupancy values of all clusters are reported in Table S1. Only in two clusters, Phe141 displays a different orientation; however, the aromatic cage is either closed or distorted. Indeed, in cluster number 8 (occupancy 4.9%), Phe141 is flipped, but it interacts with Trp151 by edge-to-face pi-pi stacking leading to a different type of closed cage conformation where the binding pocket is blocked. Instead, in cluster number 12 (occupancy 2.9%), Trp151 is totally open, and no classical aromatic cage is observed. To numerically assess the difference of the obtained clusters to the ligand-bound open cage form (PDB ID: 6I8Y, [11]), the RMSD of the aromatic cage heavy atoms were computed. The values retrieved are in the range of 1.7–3.2 Å, highlighting that the pockets attained from the MD simulation show a different conformation than that observed in to the X-ray of the ligand-bound form.

To conclude, the MD simulation of the apo-form confirmed the stability of some binding pocket residues and the flexibility of others. However, it did not generate the open conformation as observed in most ligand-bound structures, since Phe141 and Trp151 mostly interact with each other and go closer during the simulation.

### Docking and induced fit docking studies of A366

After investigating the pocket flexibility, we then tested the ability of induced fit docking (IFD) to correctly reproduce the experimentally determined X-ray binding mode of A366 (PDB ID: 6I8Y) whether an open or a closed conformation was used as starting point [11, 36, 37]. Three proteins were used: two with Phe141 in the closed cage conformation (apo-form, PDB ID: 2NS2; peptide-bound form, PDB ID: 4H75) and one with the open cage (ligand-bound form, PDB ID: 6QPL) [7, 12, 34]. Docking studies using Glide SP (rigid-body docking, protein kept rigid in its original conformation) were also performed to highlight that, in some cases, this approach can fail if there are residues in the pocket that can exhibit flexibility upon ligand binding. Thus, in these situations, treating the protein as rigid entity can be a limiting factor [38].

**Fig. 5:**
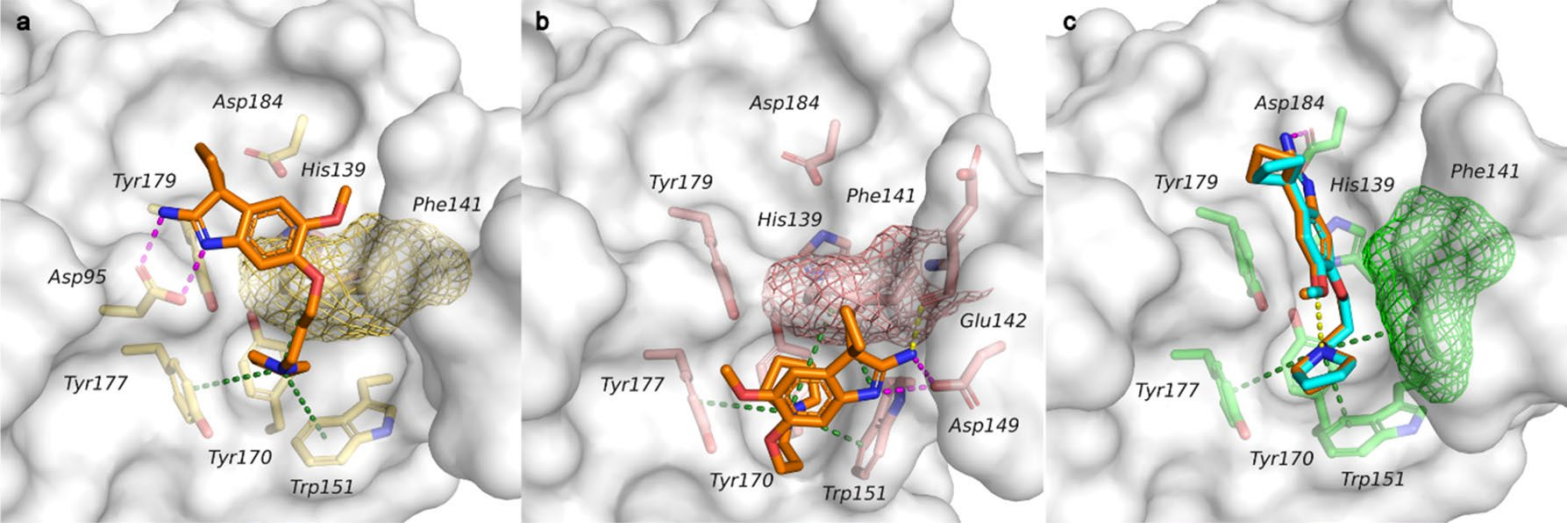
Obtained docking poses of A366 generated through Glide SP (rigid-body docking) in the second domain of diverse Spindlin1 crystal structures: **a** PDB ID: 2NS2, **b** PDB ID: 4H75, **c** PDB ID: 6QPL. The side chains of the binding pocket residues are shown as sticks, while the proteins as white surfaces. Phe141 is also illustrated as a surface mesh. Docking poses are represented as orange sticks. In **c** the experimentally determined binding mode of A366 is superimposed and displayed as cyan stick (PDB ID: 6I8Y), an RMSD of heavy atoms of 0.30 Å was observed between the obtained docking pose and the experimentally determined binding mode. Binding interactions are represented with dashed lines colored in green (cation-pi), magenta (salt bridge) and yellow (hydrogen bond)

Not surprisingly, when A366 was docked into the closed cage conformation using Glide SP, its experimentally determined binding mode (as observed in PDB ID: 6I8Y) could not be reproduced. Instead, different binding hypotheses were obtained in which the pyrrolidine moiety is always embedded in the aromatic cage and undergoes cation-pi interactions, while the core is solvent-exposed. Additional interactions with distinct residues are formed based on the orientation adopted by the ligand. As examples, the top ranked poses are illustrated in Figs. 5a and 5b. It can be noticed that the amidine moiety interacts either with Asp95 (5a) or with Asp149 and Glu142 (5b). On the other hand, when A366 was docked into the open cage conformation (not its native crystal structure), the binding interactions and the X-ray binding mode were nicely reproduced (RMSD of 0.30 Å, heavy atoms). In Fig. 5c is reported the top ranked docking pose superimposed with the X-ray ligand structure (PDB ID: 6I8Y, [11]). As in the crystal structure, salt bridge interactions between the amidine moiety and Asp184, the intramolecular hydrogen bond, as well as cation-pi interactions involving the pyrrolidine moiety and the surrounding amino acids of the aromatic cage are established.

We next performed IFD of A366 in the three selected crystal structures; apo-form (PDB ID: 2NS2), peptide-bound form (PDB ID: 4H75) and ligand-bound form (PDB ID: 6QPL). Three different IFD settings were investigated aiming at establishing a protocol that could be relatively fast and efficient. Specifically, we started by treating the seven residues that constitute the pocket as flexible; then we tested only the aromatic cage plasticity (residues: Phe141, Trp151, Tyr170, Tyr177). Since our previous structural analysis and MD simulation results clearly indicated that Phe141, Trp151 and Asp184 are the most flexible residues of the pocket, we also performed IFD where only these three residues were treated as flexible.

**Fig. 6:**
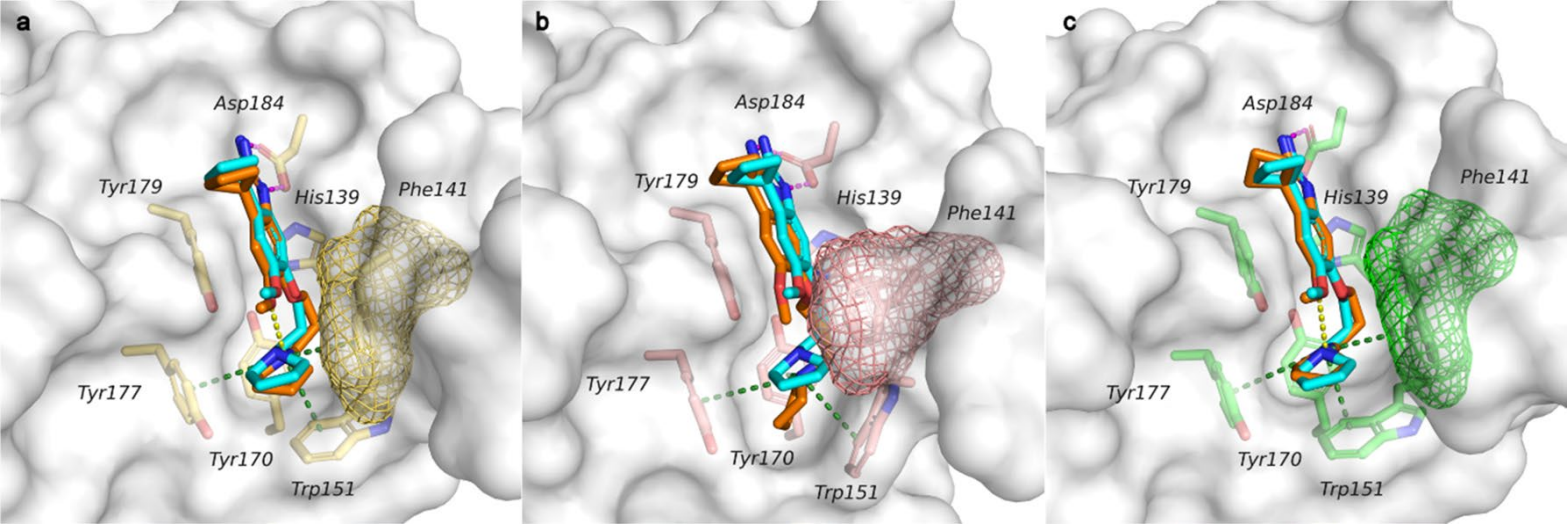
Docking poses of A366 (orange sticks) generated through IFD superimposed to the experimentally determined A366 binding mode (cyan stick, taken﻿ from PDB ID: 6I8Y). Results obtained when three residues were treated as flexible (Phe141, Trp151, Asp184). Proteins are represented as white surfaces, while the binding pocket residues are displayed as sticks, which are colored in yellow (**a** PDB ID: 2NS2), pink (**b** PDB ID: 4H75) and green (**c** PDB ID: 6QPL). Phe141 is also displayed as a surface mesh. Ligand RMSD of heavy atoms with respect to the experimentally determined A366 binding mode: **a** 0.61 Å; **b** 1.45 Å; **c** 0.48 Å. Salt bridges, hydrogen bonds and cation-pi interactions are represented as dashed lines, in magenta, yellow and dark green, respectively

The three different IFD settings and proteins yielded docking poses that could very nicely reproduce the binding interactions and the X-ray pose of A366 with low RMSD values (< 1.8 Å, heavy atoms). In the Supporting Information (Figure S3) the top ranked poses retrieved when either seven or four amino acids were treated as flexible are reported. Meanwhile, the poses obtained by treating three residues as flexible are shown in Fig. 6 and discussed below.

Interestingly, the flip of the Phe141 was always induced by A366. When the apo-form was used as starting conformation, a pose with a perfect overlap to the experimentally determined binding mode was generated (RMSD of 0.61 Å, heavy atoms; Fig. 6a). Besides the salt bridge and cation-pi interactions, the intramolecular hydrogen bond between the NH^+^ of the positively charged pyrrolidine moiety and the methoxy group is also observed. We then tested the peptide-bound form conformation as starting point. The IFD protocol generated good results with an RMSD of 1.45 Å (heavy atoms) for the top ranked pose (Fig. 6b). However, some deviations from the experimentally observed binding mode of A366 could be detected. The pyrroline moiety which is still placed in the aromatic cage is more tilted, but the linker group shows a more extended conformation, and the methoxy group is differently orientated. Consequently, the intramolecular hydrogen bond interaction between the pyrrolidine NH^+^ and the methoxy group cannot be formed. Noteworthy, the role of this intramolecular interaction has been investigated by the design and biological testing of A366 analogs that miss the intramolecular hydrogen bond and that are no longer active (data not shown, data will be published elsewhere). It is worth noting that when IFD was applied to the open cage structure, the open conformation was maintained and the binding mode was reproduced as observed in the X-ray (RMSD of 0.48 Å, heavy atoms; Fig. 6c).

As described in the next section, the docking poses obtained by IFD were further investigated by running short MD simulations. We specifically wanted to investigate whether the obtained IFD pose in the peptide-bound structure PDB ID 4H75 (Fig. 5b) could be optimized and stabilized into the experimentally determined binding mode by running a short MD simulation. Furthermore, the stability of the predicted binding modes attained in the apo-form (PDB ID: 2NS2) through rigid-body docking (Glide SP) as well as IFD was also verified by means of MD simulations.

### Analysis of the predicted binding modes by MD simulations

**Fig. 7:**
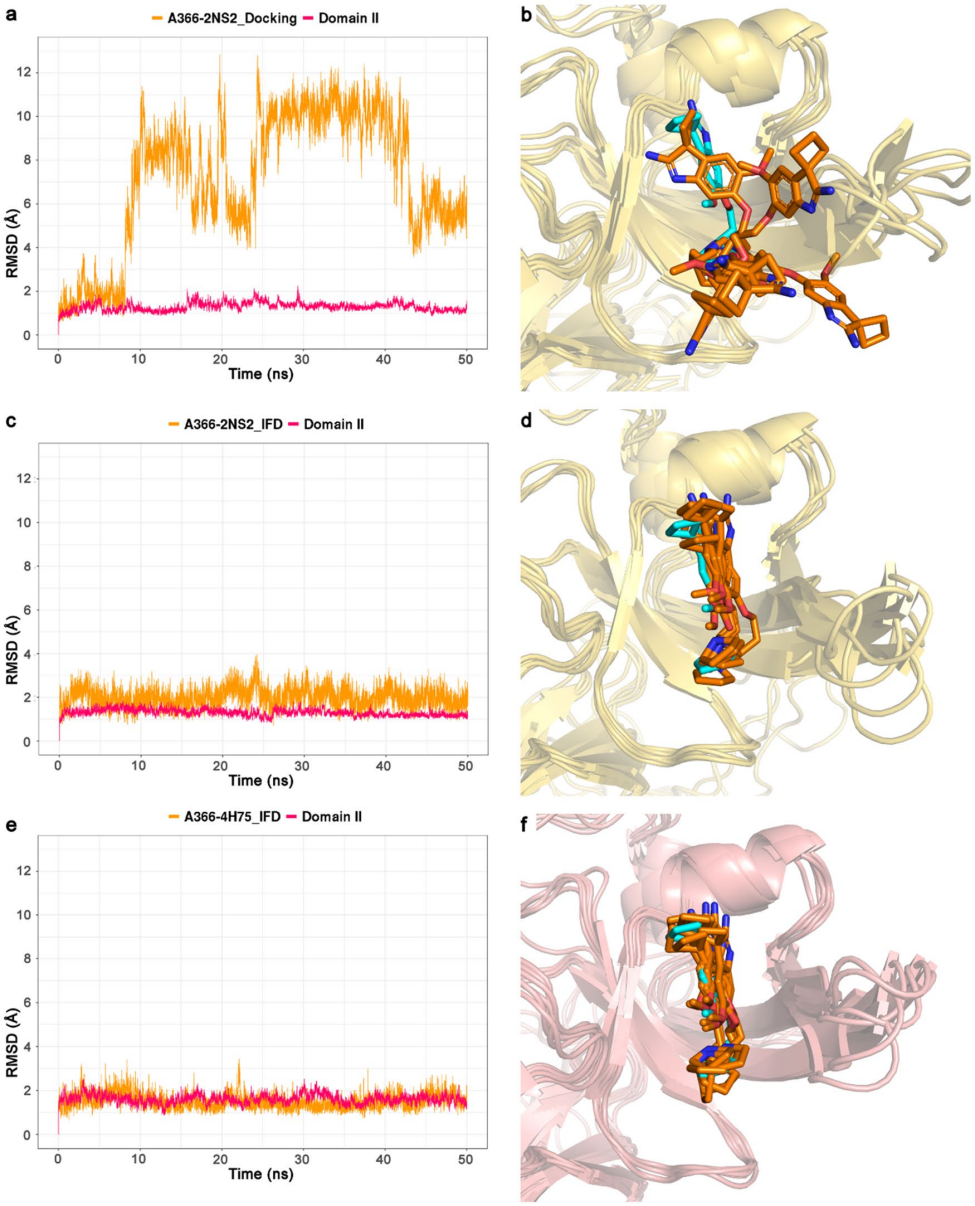
Analysis of 50 ns MD simulations of A366-Spindlin1 complexes obtained from rigid-body docking with Glide SP and IFD using two protein structures (PDB IDs: 2NS2 and 4H75; represented in yellow and pink, respectively). **a**, **b** A366-2NS2_Docking; **c**, **d** A366-2NS2_IFD; **e**, **f** A366-4H75_IFD. In **a**, **c**, **e** are plotted the root mean square deviation (RMSD) values of the proteins (Domain II, backbone atoms) and ligand (heavy atoms). In **b**, **d**, **f** are shown the binding modes of A366 observed during the simulations at 0 ns, 10 ns, 20 ns, 30 ns, 40 ns and 50 ns (orange sticks) superimposed with the experimentally determined X-ray ligand structure (cyan stick, PDB ID: 6I8Y)

**Fig. 8:**
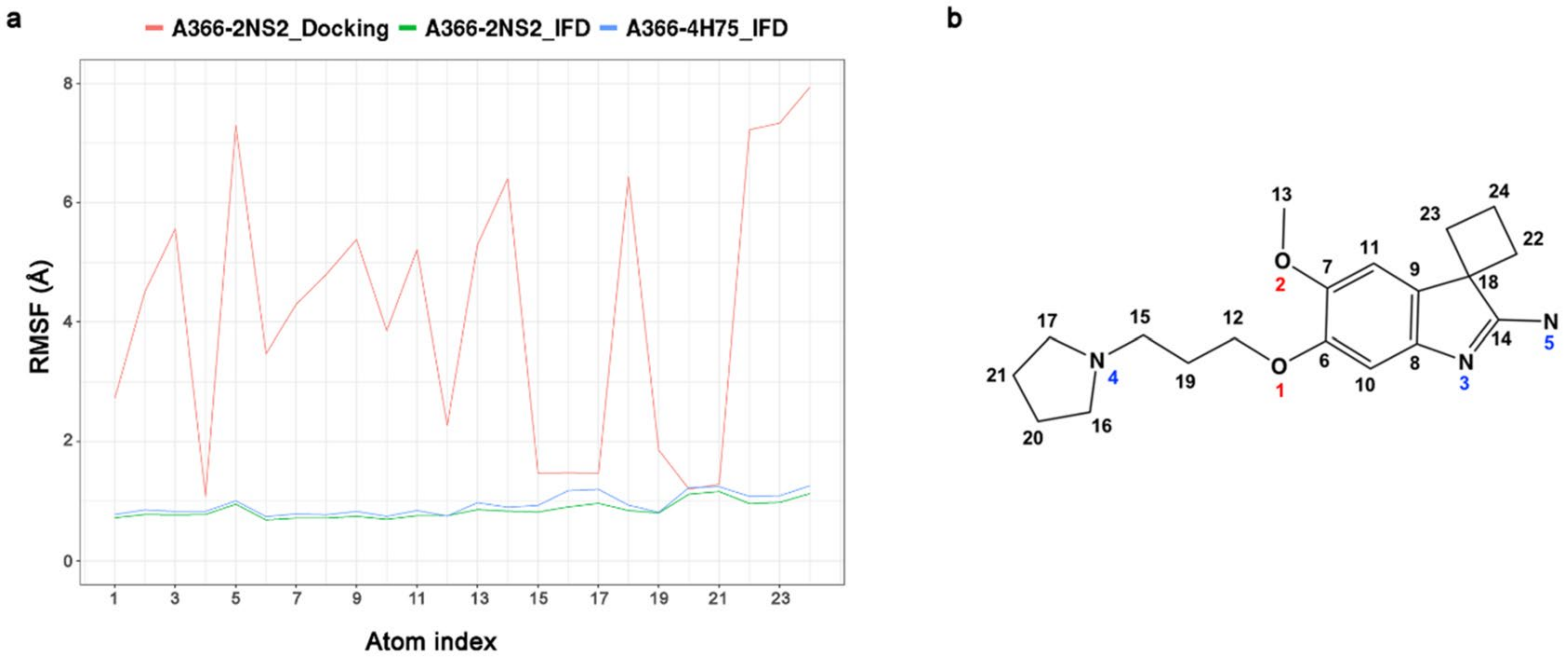
Ligand root mean square fluctuations (RMSF) values retrieved from 50 ns MD simulations of A366-Spindlin1 complex obtained from rigid-body docking with Glide SP and IFD using two protein structures (PDB IDs: 2NS2 and 4H75). In **a** are plotted the ligand’s fluctuations broken down by atom, whereas in **b** A366 2D structure with the corresponding atom index is shown

To verify the stability of the predicted binding modes obtained from rigid-body docking (Glide SP) and IFD, the retrieved poses-complexes were subjected to MD simulations using Desmond package [31]. Specifically, we wanted to investigate if the binding mode were stable during the MD simulations and in line with the experimentally determined binding mode of A366. Moreover, since the pose attained from 4H75 with IFD did not show the intramolecular hydrogen bond, we tested if the binding pose could be optimized by running a short MD simulation. The docking results reported in Fig. 5a (A366-2NS2_Docking), Fig. 6a (A366-2NS2_IFD) and Fig. 6b (A366-4H75_IFD) were used as initial coordinates for the generation of the MD systems. The analysis of the simulations was focused primarily on the binding mode stability, thus, RMSD and RMSF values were calculated and plotted in Fig. 7 and Fig. 8, respectively.

The analysis of the MD simulation of rigid-body docking of A366 in the apo-form (A366-2NS2_Docking, Fig. 5a) highlighted that the binding mode predicted into the closed aromatic cage is highly unstable during the simulation (Fig. 7a). While the pyrrolidine moiety remains in the cage, the core, which is more solvent exposed, fluctuates and generates diverse binding modes (Fig. 7b). The RMSD values are indeed very high (Fig. 7a) as well as the RMSF of the majority of the ligand atoms (Fig. 8). Phe141 does not flip during the simulation and the experimentally determined pose of A366 is not reproduced.

Meanwhile the obtained IFD pose of A366 in the apo-form structure (A366-2NS2_IFD), which showed a binding mode that perfectly reproduces the experimentally A366 X-ray conformation (Fig. 6a), is highly stable during the MD simulation with the initial pose being maintained throughout the simulation time (Fig. 7c and 7d). The intramolecular hydrogen bond is preserved (occupancy rate of 78.8%), and only marginal fluctuations of the ligand atoms are detected (Fig. 8).

Finally, the MD simulation performed for the IFD pose of A366 in the peptide-bound structure (A366-4H75_IFD, Fig. 6b) showed that the ligand is quickly adopting the binding conformation observed in the A366 crystal structure (Fig. 7f). The pyrrolidine moiety and the methoxy group orientate themselves to form the intramolecular hydrogen bond which is further conserved during the simulation (occupancy rate of 73.6%). The initial IFD binding pose is optimized and minimal fluctuations of the ligand atoms are noticed (Fig. 8).

Hence, these results clearly demonstrate that either IFD alone or IFD combined with a short MD simulation can be used to reproduce the experimentally binding mode of A366 starting from closed aromatic cage conformations.

## Conclusions

Through in silico methods, we investigated the Spindlin1 aromatic cage plasticity and the ability of different methods to correctly reproduce the experimentally determined binding mode of A366. The idea behind the present work was to probe how flexible docking performs using different protein conformations as starting points, especially in the case where pocket flexibility is known to occur such as in methyl-lysine reader proteins.

Several studies have suggested that using clusters obtained from MD as initial coordinates for further docking studies can help to address the protein flexibility, a major limitation of rigid-body docking [39–43]. In the herein reported case, however, classical MD simulation studies clearly failed to generate a binding pocket conformation, which would be suitable for the ligand binding. Only closed cage conformations or disorganized cage were observed throughout the MD simulation time (both 50 and 500 ns), which clearly hinder the binding of A366 in the correct conformation. The failure of the MD simulation to reproduce the open cage conformation, as observed in most ligand-bound structures, can be attributed to the hydrophobic nature of the aromatic cage, where, in the absence of any ligand, Phe141 is driven by pi-pi stacking interactions towards Trp151, leading to the aromatic cage being mainly stuck in the closed conformation.

Meanwhile, IFD in various pocket conformations was generally able to generate highly satisfactory results. The open cage conformation was generated upon A366 binding and the obtained docking poses could nicely reproduce the X-ray ligand binding mode of A366 showing low RMSD values as low as 0.61 Å even when starting with the closed cage conformation. Noteworthy, prior analysis of the crystal structures could shed light on the binding pocket flexibility to guide the selection of the amino acids for the IFD.

Short MD simulation (50 ns) on the obtained docking poses also proved to be very helpful to verify the obtained binding modes by analysing their stability. Indeed, the obtained docking pose in the closed cage conformation of Spindlin1, which is clearly incorrect as demonstrated by the resolved crystal structure in complex with A336, is plainly unstable during the MD simulation. Meanwhile, binding modes where A366 is embedded in an open cage conformation, which replicate the experimentally determined binding mode, show high stability during the MD simulation. Furthermore, short MD simulations (50 ns) could help to “fine-tune” the predicted IFD binding mode by optimizing the interactions, as observed for the IFD pose of A366 in the peptide-bound structure.

The reported approach of IFD followed by short MD simulations of the obtained binding modes is a highly promising combination to rightly predict the binding mode of small molecule ligands in flexible binding pockets, such as observed in reader proteins. The protocol might be useful to discover novel small molecule ligands for the yet unexplored reader proteins.

## Experimental methods

### Protein preparation

Several Spindlin1 crystal structures available in the Protein Data Bank (PDB; www.rcsb.org) [29] were downloaded and prepared with Schrödinger’s Protein Preparation Wizard tool [44]. Following the PDB IDs of the investigated structures: 2NS2, 4H75, 4MZF, 5Y5W, 5JSG, 5JSJ, 6I8Y, 6QPL [7, 8, 10–13, 34]. Solvent molecules, except the water molecules present in the second domain, and sodium ions were removed. Hydrogen atoms, missing side chain residues and loops were added to the protein structures. Afterward, protonation states were assigned with PROPKA at pH 7.0 and the hydrogen bonding networks were optimized. Finally, the protein structures were energy-minimized using the OPLS3 force field and default settings.

### Analysis of protein structures

The prepared crystal structures were analyzed in Maestro [45]. First, the co-crystallized histones and ligands were removed for clarity. Then, the protein structures were superimposed with the Protein Structure Alignment tool and the protein residues were colored by B-factor. Attention was given to the aromatic cage residues.

### Ligand preparation

A366 structure was drawn by means of Maestro 2D sketcher [37] and was then prepared with Schrödinger’s LigPrep tool [46]. All possible tautomeric forms and stereoisomers were generated at pH 7.0 ± 1.0 using Epik. Next, ConfGen was employed for the generation of a multi-conformational dataset: a maximum of 50 conformers was allowed, and the output conformations were energy-minimized using the default force field (OLPS_2005) [47, 48]. All conformers were used as input for rigid-body docking (Glide SP), whereas the lowest energy conformation was selected for flexible docking (IFD).

### Docking studies: rigid-body docking (Glide SP) and flexible docking (IFD)

Three previously prepared crystal structures were selected for docking studies: two with Phe141 in the closed cage conformation (apo-form, PDB ID: 2NS2; peptide-bound form, PDB ID: 4H75) and one with the open cage (ligand-bound form, PDB ID: 6QPL) [7, 12, 34]. One water molecule in each protein was kept (2NS2: HOH363, 4H75: HOH416, 6QPL: HOH425) and considered in the docking procedure. The grid boxes were prepared by assigning Phe141 as the centroid and a cube of 15 Å was defined as the inner box.

#### Rigid-body docking (Glide SP)

Molecular docking studies were carried out with Glide using the Standard Precision (SP) mode [35]. In this approach, the protein is maintained rigid in its original conformation, whereases ligands are treated as flexible by default. Within this work, we refer to such method as rigid-body docking. The options "sample ring conformation" and "reward intramolecular hydrogen bonds” were switched on and a maximum of three docking poses were output for each conformer; all other settings were kept as default. The predicted binding modes were analyzed by visual inspection of the top-scored poses.

#### Flexible docking (IFD)

Flexible docking studies were performed through Induced Fit Docking (IFD) [32]. Different settings were tested; specifically, the Extended Sampling protocol was chosen, and diverse sets of residues to be refined with Prime were examined. The ligand was always treated as flexible and sample ring conformations option was selected; all other settings were left as default. The final results reported here refer to three protocols which encompassed diverse combinations of residues treated as flexible: i) seven residues that constitute the pocket (His139, Phe141, Trp151, Tyr170, Tyr177, Tyr179, Asp184); ii) aromatic cage (Phe141, Trp151, Tyr170, Tyr177); iii) three amino acids (Phe141, Trp151, Asp184). The predicted binding modes were analyzed by visual inspection of the top-scored poses.

### Molecular dynamics simulations

Four different MD simulations were run. Initially, the apo-protein (PDB ID: 2NS2, [34]) was explored in order to investigate the flexibility of the aromatic cage. Later, MD simulations of the predicted binding modes obtained from rigid-body docking with Glide SP (PDB ID: 2NS2) and IFD (PDB ID: 2NS2 and 4H75) were carried out to analyze their stability [7, 34]. Thus, the following structures were used as initial coordinates for the generation of the MD systems: 2NS2 as apo-form, rigid body docking as well as IFD pose of A366 in 2NS2, IFD pose of A366 in 4H75. The top-ranked docking poses were taken from the Glide SP docking and IFD studies described above. Desmond software suite was employed to set up the systems and run the MD simulations [31]. The systems were solvated using the TIP3P water model in a Periodic Boundary Conditions orthorhombic box of 10 Å and neutralized with Na^+^ ions at a salt concentration of 0.15 M. For all simulations, the OPLS3 force field and NPT (temperature (T), pressure (P), and the number of particles (N)) ensemble was used. Before performing the production simulation, the default Desmond protocol for energy minimization and model relaxation were utilized. Finally, 50 ns MD simulations with a trajectory interval of 5 ps were carried out at a temperature of 300° K in the NPT ensemble using a Nose–Hoover chain thermostat and a Martyna-Tobias-Klein barostat (1.01325 bar). For the second MD simulation of 2NS2 apo-form system the time was extended to 500 ns.

For the analysis of the MD simulations, three Schrödinger’s tools were used: Simulation Interactions Diagram (SID), Simulation Event Analysis (SEA) and Desmond trajectory clustering script [31]. SID was employed to generate the ligand’s root mean square fluctuations (RMSF) and root mean square deviation (RMSD). Meanwhile, SEA was used to obtain the RMSD and RMSF values for the proteins and the occupancy rates of the investigated hydrogen bonds among the protein residues and the ligand intramolecular hydrogen bond. The retrieved values were then plotted using R package. The Desmond trajectory clustering script was used to cluster the MD simulation frames of 2NS2 apo-form based on the RMSD matrix of Phe141 (heavy atoms).

## Supplementary Information

Below is the link to the electronic supplementary material.

Supplementary file1 (DOCX 2710 kb)
