# Supplementary material for: Exploring aromatic cage flexibility of the histone methyllysine reader protein Spindlin1 and its impact on binding mode prediction: an in silico study
Source: J Comput Aided Mol Des. 2021 Jun 3;35(6):695–706. doi: 10.1007/s10822-021-00391-9 (PMC8213585; doi:10.1007/s10822-021-00391-9)
Supplement: Supplementary file 1 — Supplementary file1 (DOCX 2710 kb) [file 10822_2021_391_MOESM1_ESM.docx]

**Supporting Information**


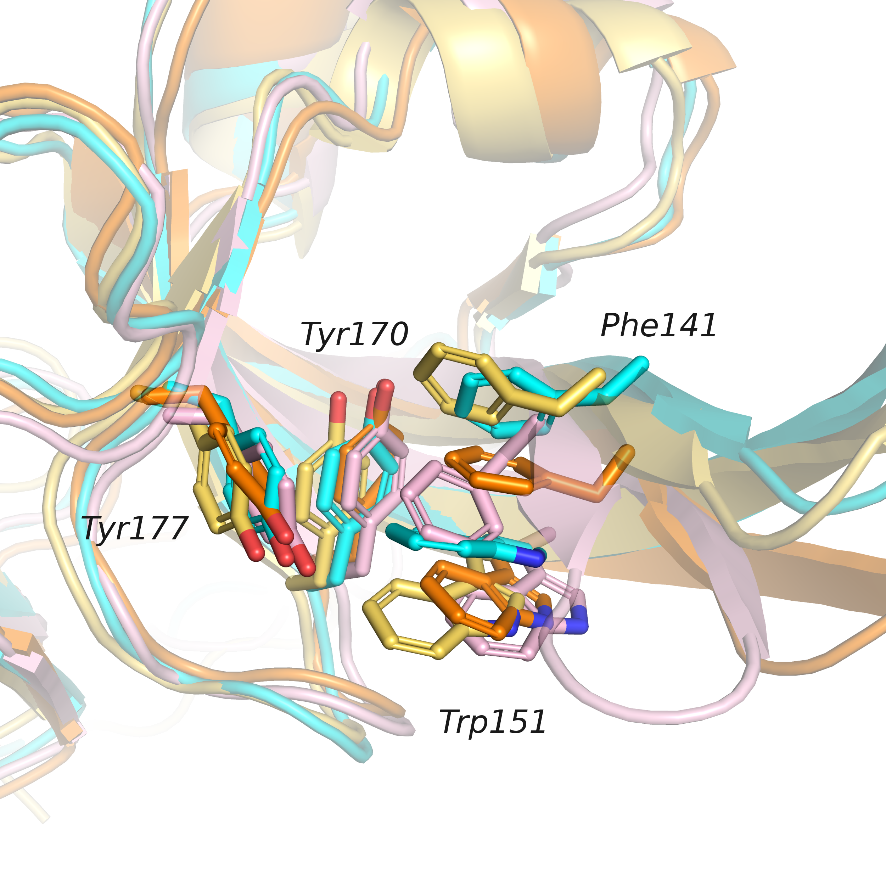


**Figure S1.** Superimposition of representative frames for each of the three clusters obtained from the clustering analysis of the 50 ns MD simulation. Following cluster number, displayed colors and occupancy rates: i) cluster 1, orange, 82.2%; ii) cluster 2, cyan, 15.4 %; iii) cluster 3, light pink, 2.4%. The reference crystal structure is also displayed in yellow (PDB ID: 2NS2).

**
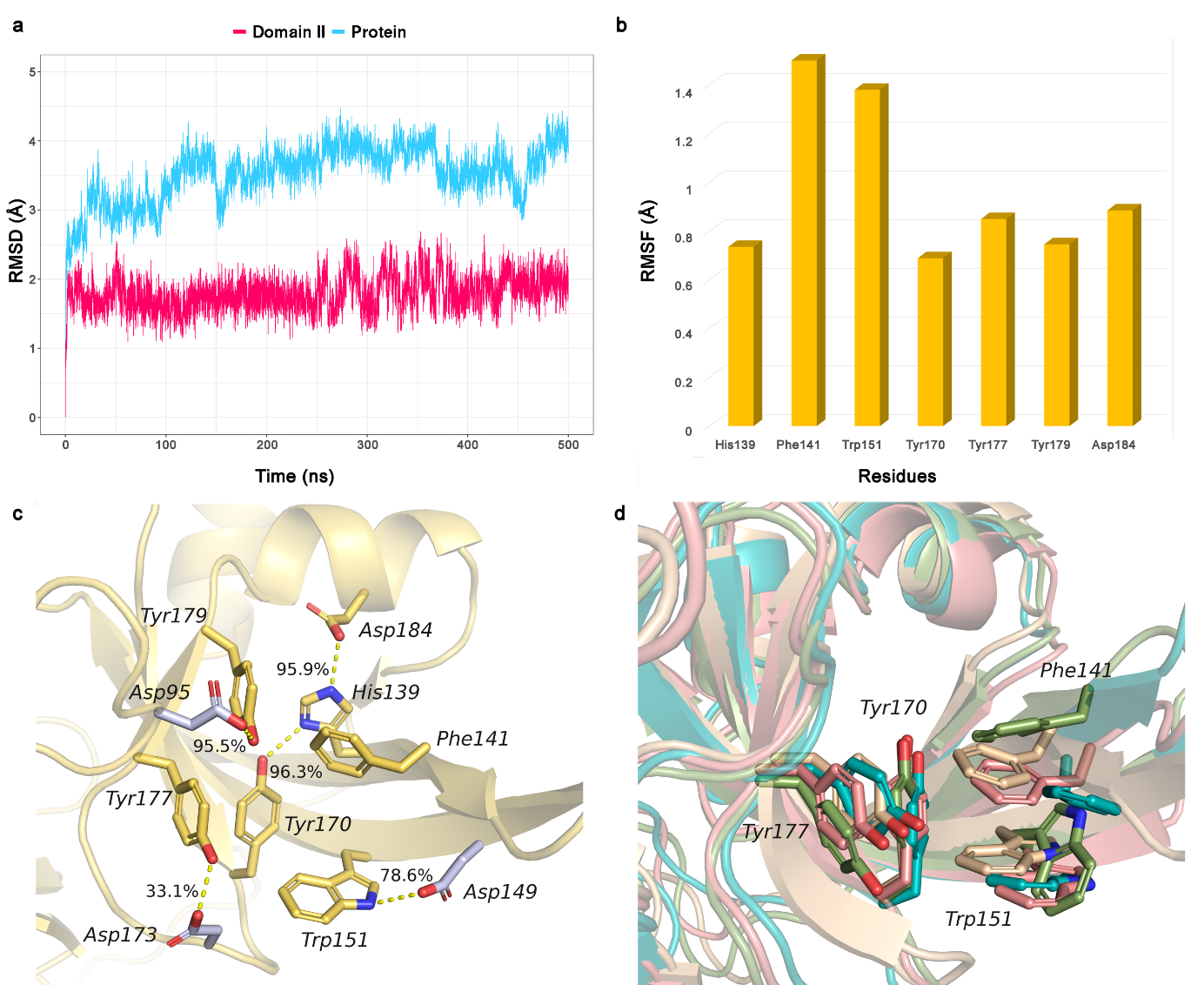
**

**Figure S2.** Analysis of 500 ns MD simulation of the apo-form Spindlin1 (PDB ID: 2NS2). (**a**) Root mean square deviation (RMSD) plots of the backbone atoms. (**b**) Root-mean-square fluctuation (RMSF) values of the binding pocket residues, heavy atoms. (**c**) Binding pocket residues (yellow sticks) and hydrogen bonds (yellow dashed lines) as observed in the crystal structure (PDB ID: 2NS2); occupancy values of the interactions during the MD simulation. (**d**) Superimposition of representative frames for each of the first four clusters obtained from the clustering analysis. Following cluster number, displayed colors and occupancy rates: i) cluster 1, pink, 24.3%; ii) cluster 2, dark cyan, 10.0 %; iii) cluster 3, dark green, 8.4%; iv) cluster 4, beige, 7.9%.

**Table S1.** Clustering analysis of the 500 ns MD simulation.

| Cluster number | Occupancy rate  (%) |
| --- | --- |
| 1 | 24.3 |
| 2 | 10.0 |
| 3 | 8.4 |
| 4 | 7.9 |
| 5 | 7.8 |
| 6 | 7.5 |
| 7 | 7.2 |
| 8 | 4.9 |
| 9 | 4.7 |
| 10 | 4.7 |
| 11 | 4.0 |
| 12 | 2.9 |
| 13 | 2.1 |
| 14 | 1.5 |
| 15 | 1.4 |
| 16 | 0.8 |


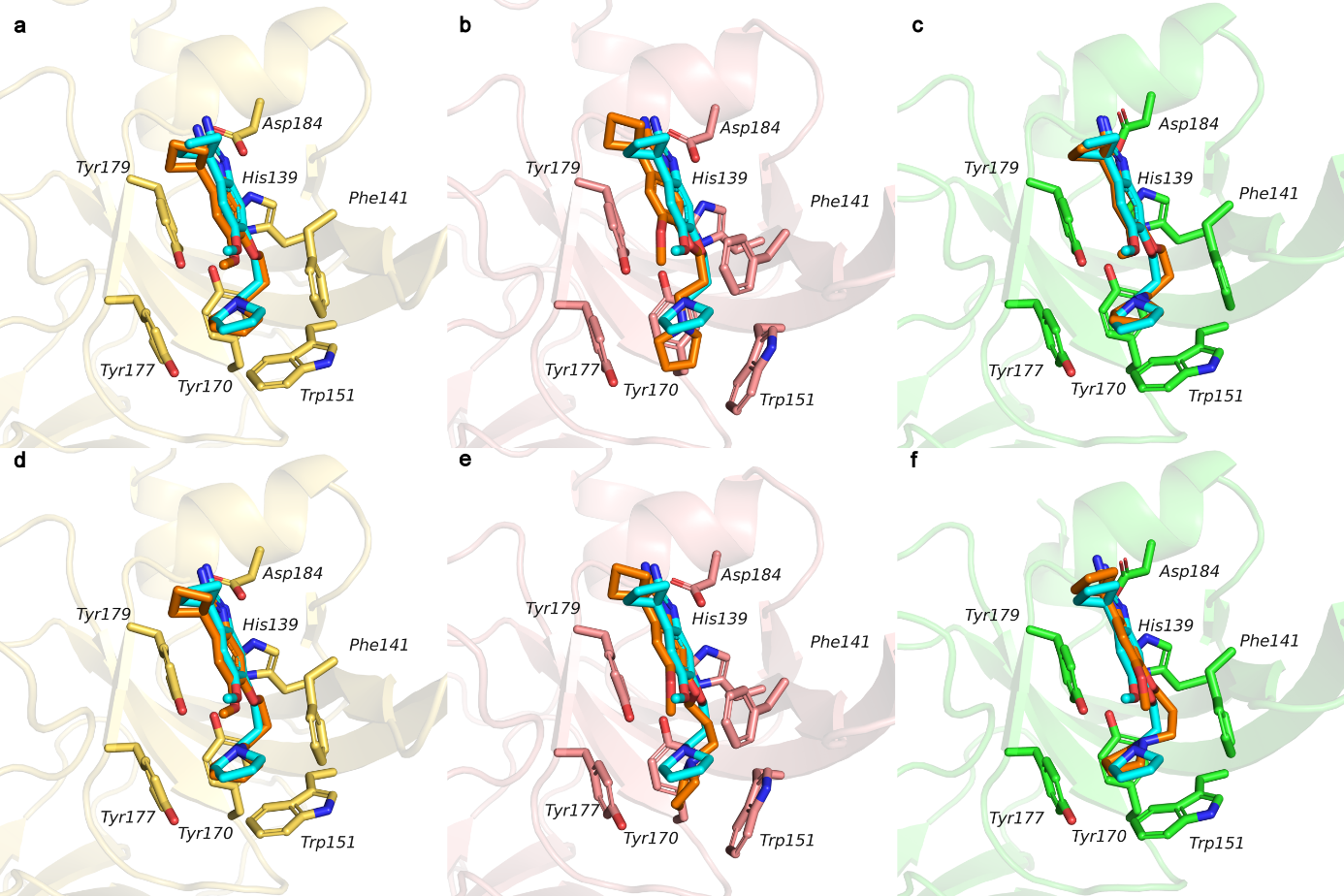


**Figure S3.** Binding modes of A366 generated through IFD (orange sticks) superimposed to the experimentally determined X-ray structure (cyan stick, PDB ID 6I8Y). In sticks are depicted the binding pocket residues, which are colored in yellow (PDB ID: 2NS2), pink (PDB ID: 4H75) and green (PDB ID: 6QPL). In (a) (b) (c) are represented the results obtained when all seven residues displayed are treated as flexible, while in (d) (e) (f) the ones when only the four aromatic residues of the cage are treated as flexible (Phe141, Trp151, Tyr170, Tyr177). Ligand RMSD of heavy atoms with respect to the experimentally observed binding mode of A366: (a) 0.32 Å; (b) 1.73 Å; (c) 0.50 Å; (d) 0.68 Å; (e) 1.35 Å; (f) 1.38 Å.
